# Supplementary material for: Enhanced B Cell Receptor Signaling Partially Compensates for Impaired Toll-like Receptor 4 Responses in LPS-Stimulated IκBNS-Deficient B Cells
Source: Cells. 2023 Apr 24;12(9):1229. doi: 10.3390/cells12091229 (PMC10177494; doi:10.3390/cells12091229)
Supplement: Supplementary file 1 [file cells-12-01229-s001.zip › cells-2284432-supplementary.pdf]

cells

MDPI

Supplementary Materials

Enhanced B Cell Receptor Signaling Partially Compensates for Impaired Toll-Like Receptor 4 Responses in LPS-Stimulated IκBNS-Deficient B Cells

Monika Adori[1], Sharesta Khoenkhoen[1], Jingdian Zhang[2], Xaquin Castro Dopico[1] and Gunilla B. Karlsson Hedestam[1,*]

1. Department of Microbiology, Tumor and Cell Biology, Karolinska Institutet, 171 77 Stockholm, Sweden
2. Department of Medical Biochemistry and Biophysics, Division of Molecular Metabolism and Karolinska Institutet, 171 77 Stockholm, Sweden

* Correspondence: gunilla.karlsson.hedestam@ki.se

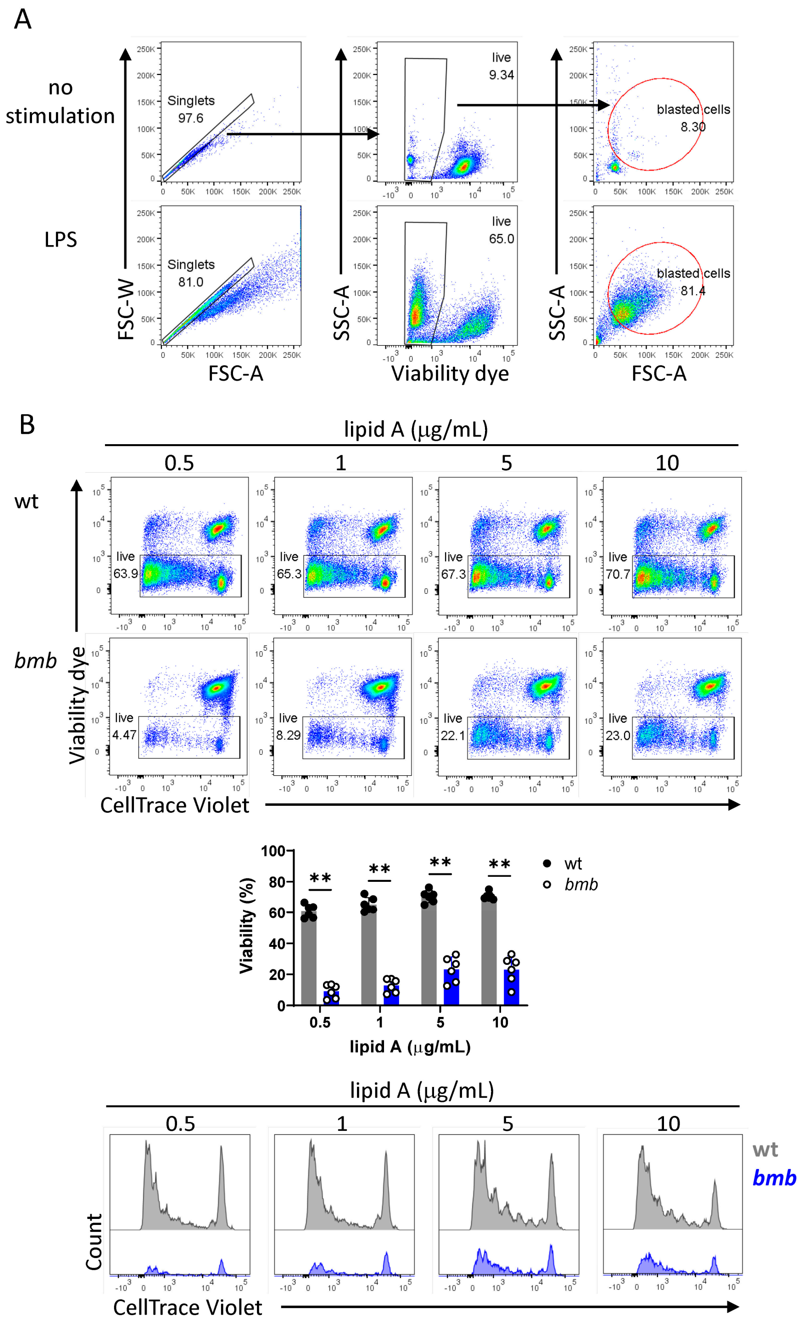

Figure S1. Impaired cell viability and altered B cell proliferation to lipid A in IκBNS-deficient *bumble* mice. (**A**) Representative flow cytometry plots showing the gating strategy for blasting B cells from wt mice stimulated with 10 μg/mL LPS or left unstimulated for 72 hours. (**B**) Viability and cell proliferation were assessed after 72 h lipid A stimulation. Representative flow cytometry plots showing co-staining of CellTrace Violet (CTV) and fixable live/dead dye after 3 days activation. Numbers adjacent to the gates show live cell frequency in the parent (singlets) population. Bar graph depicts frequency of live cells (viability dye^{low}) for each concentration. Representative overlayed histograms showing cell divisions for each lipid A concentration, as indicated, from wt and *bmb* mice. Statistical differences between strains calculated by Mann–Whitney *t*-test, ** for $p \leq 0.01$.

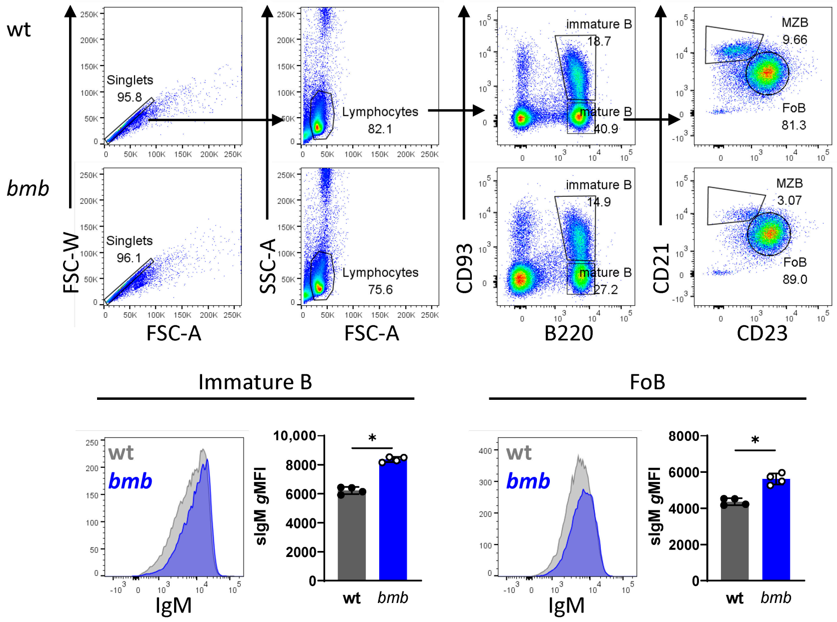

Figure S2. Elevated surface IgM expression on IκBNS-deficient immature B and mature FoB cells. Representative flow cytometry plots showing gating strategy for immature and mature (MZB and FoB) B cell populations of freshly isolated splenocytes from wt and IκBNS-deficient *bumble* (*bmb*) mice. Numbers adjacent to the gates mark cell frequency in the parent population. Representative overlayed histograms showing surface IgM expression on immature B and FoB cells from wt (grey) and *bmb* (blue) mice. Adjacent bar graphs show geometric mean fluorescence intensity (*g*MFI) for IgM in wt (grey) and *bmb* (blue) cells. Significant differences between strains were calculated by Mann-Whitney *t*-test, * for $p \leq 0.05$.

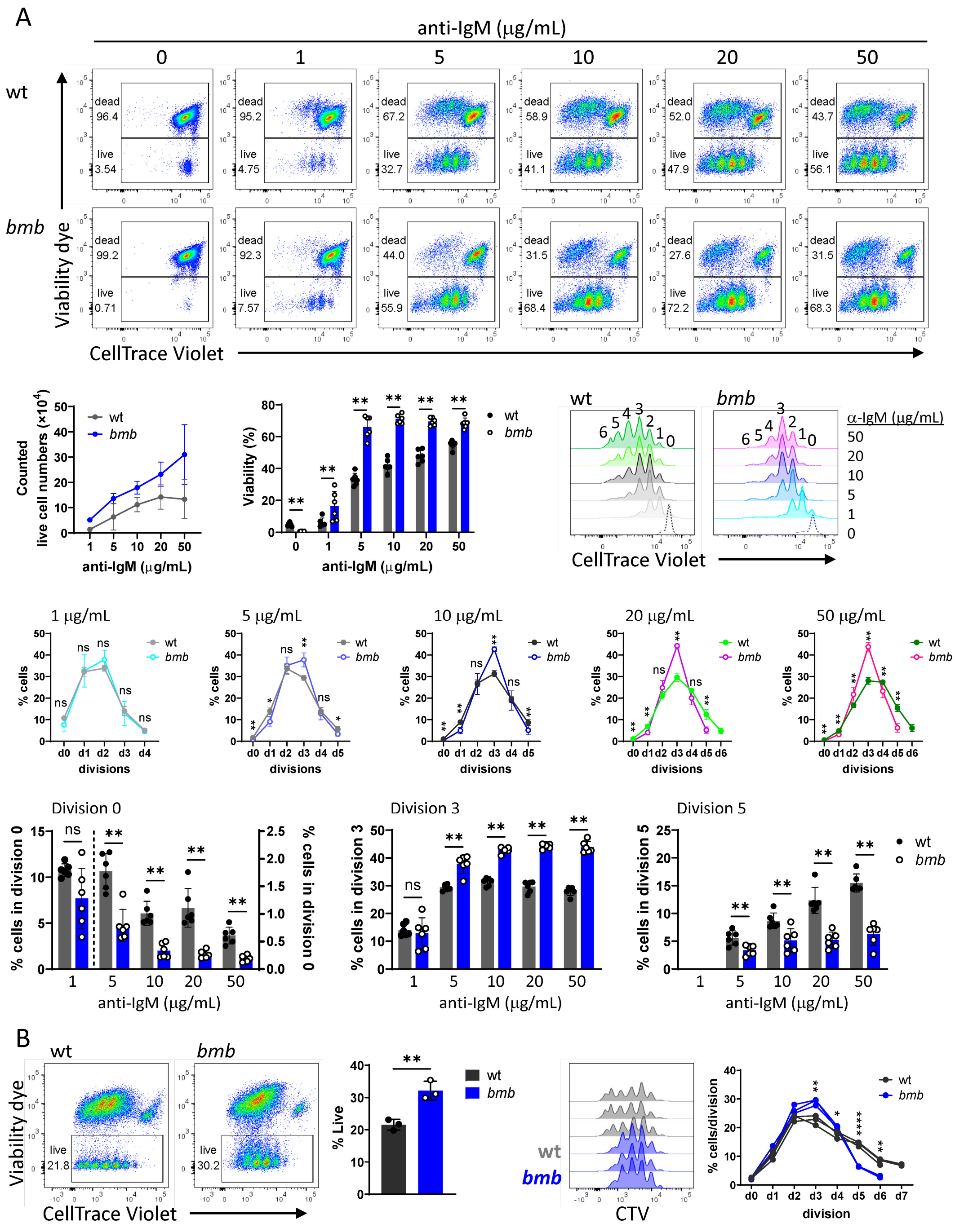

Figure S3. Normal B cell proliferation to anti-IgM stimulation requires IκBNS. CTV-labeled B cells from wt and IκBNS-deficient *bumble* (*bmb*) mice were stimulated with anti-IgM (Fab′)₂, and viability and cell proliferation were assessed. (**A**) wt or *bmb* B cells were stimulated with various concentrations of anti-IgM, as indicated. Representative flow cytometry plots show double staining of CellTrace Violet (CTV) and fixable live/dead dye after 3 days activation. Lower left graph depicts counted live cell numbers for each concentration. Bar graphs in the middle show frequencies of viable (viability dye^{low}) cells. Representative overlayed histograms show cell divisions for each anti-IgM concentration from wt and *bmb* mice. (**B**) B cells from wt and *bmb* mice were CTV labeled and stimulated *in vitro* with 10 μg/mL anti-IgM for 5 days. Flow cytometry plots represent CTV and viability dye co-stained cells from wt and *bmb*. Adjacent bar graphs show frequency of live cells in wt (grey) and *bmb* (blue) cultures. Overlayed histograms show CTV dilutions of live cells in wt (grey) and *bmb* (blue) cell cultures. Frequencies of cells per divisions were determined. Data in (**A**) are representative of two independent experiments, with 3 or 6 mice. Significant differences

between strains were calculated by Mann–Whitney *t*-test, * for $p \leq 0.05$ (**A**), or by unpaired *t*-test, ns (non-significant) for $p > 0.05$, * for $p \leq 0.05$, ** for $p \leq 0.01$, **** for $p \leq 0.0001$ (**B**).

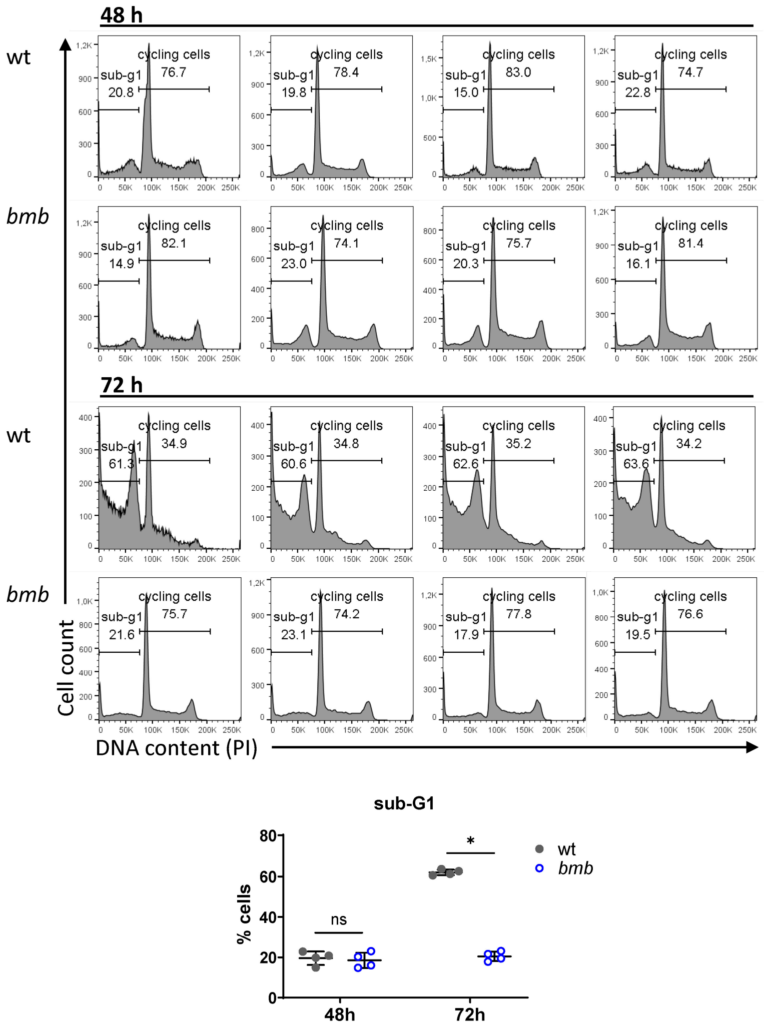

Figure S4. Sub-G1 population in anti-IgM stimulated IκBNS-deficient B cell culture. B cells from wt and *bmb* mice were stimulated with 10 µg/mL anti-IgM for 48 and 72 h and DNA content were examined by PI staining. Histograms showing PI fluorescence intensities. Numbers adjacent to the gates indicate cell frequency in the parent population (single cells). Graphs show statistical analysis for sub-G1 frequencies in wt (grey) and *bmb* (blue). Data are representative of two independent experiments, with 3 or 4 mice for each strain. Significant differences were calculated by Mann–Whitney *t*-test, ns (non-significant) for $p > 0.05$, * for $p \leq 0.05$.

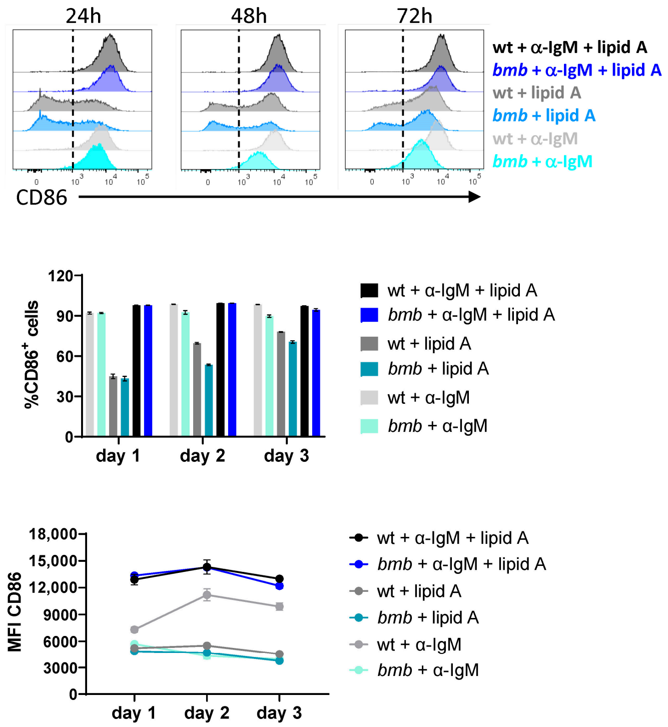

Figure S5. Synergistic effect of combined anti-IgM and lipid A stimuli on CD86 expression in IκBNS-deficient B cells. B cells from wt and *bmb* mice were stimulated with 10 µg/mL anti-IgM, 1 µg/mL lipid A, or a combination of both, for 24, 48, and 72 h, and CD86 expression was analyzed by flow cytometry. Histograms showing CD86 fluorescence intensities. Bar graphs show frequencies of CD86+ cells. Lower graphs showing mean fluorescence intensity (MFI) values for CD86 in the CD86+ population. Data are representative of two independent experiments, with 3 or 4 mice for each strain.
